# The Genetics of Vitamin C Loss in Vertebrates

**DOI:** 10.2174/138920211796429736

**Published:** 2011-08

**Authors:** Guy Drouin, Jean-Rémi Godin, Benoît Pagé

**Affiliations:** Département de Biologie et Centre de Recherche Avancée en Génomique Environnementale, Université d'Ottawa, Ottawa, Ontario, K1N 6N5, Canada

**Keywords:** Ascorbic acid, biosynthesis, *GLO* gene, L-gulono-gamma-lactone oxidase, pseudogene, vitamin C.

## Abstract

Vitamin C (ascorbic acid) plays important roles as an anti-oxidant and in collagen synthesis. These important roles, and the relatively large amounts of vitamin C required daily, likely explain why most vertebrate species are able to synthesize this compound. Surprisingly, many species, such as teleost fishes, anthropoid primates, guinea pigs, as well as some bat and Passeriformes bird species, have lost the capacity to synthesize it. Here, we review the genetic bases behind the repeated losses in the ability to synthesize vitamin C as well as their implications. In all cases so far studied, the inability to synthesize vitamin C is due to mutations in the L-gulono-γ-lactone oxidase (*GLO*) gene which codes for the enzyme responsible for catalyzing the last step of vitamin C biosynthesis. The bias for mutations in this particular gene is likely due to the fact that losing it only affects vitamin C production. Whereas the *GLO* gene mutations in fish, anthropoid primates and guinea pigs are irreversible, some of the *GLO* pseudogenes found in bat species have been shown to be reactivated during evolution. The same phenomenon is thought to have occurred in some Passeriformes bird species. Interestingly, these *GLO* gene losses and reactivations are unrelated to the diet of the species involved. This suggests that losing the ability to make vitamin C is a neutral trait.

## INTRODUCTION

Vitamins are organic compounds which have to be obtained from the diet, either because an organism does not have the enzymes necessary to synthesize them or because it cannot produce them in sufficient quantities [1]. Humans cannot synthesize vitamins A, B1 (thiamine), B2 (riboflavin), B5 (pantothenic acid), B6 (pyridoxine), B7 (biotin), B9 (folate), B12 (cobalamin), E and K but are able to synthesize some vitamin B3 (niacin) and D. The last vitamin required by humans, vitamin C (ascorbic acid), is a special case in that this organic compound is synthesized by the large majority of vertebrate and invertebrate species [2-5]. Interestingly, the vertebrate organ used to synthesize vitamin C changed twice from the kidney to the liver during evolution, once in birds and once in mammals. Whereas vitamin C is produced by the kidneys of fishes, amphibians, reptiles, and older bird orders, it is produced by the liver of more recent bird orders and of mammals [6]. This switch to a larger organ in more active species has been interpreted as being the result of selective pressures to maintain biochemical homoeostasis under more stressful conditions [2, 7]. This is reflected by the fact that the human daily recommended intake for vitamin C (60 mg) is the highest among all vitamins [8, 9]. 

Vitamin C is a water-soluble compound with anti-oxidant properties that protects living organisms against oxidative stress [10]. It is also essential for collagen synthesis, which is why insufficient amounts of this vitamin lead to scurvy [11]. Its other functions include the synthesis of carnitine, neurotransmitters and the catabolism of tyrosine, among others [1, 12, 13].

Yeasts, plants and animals use different pathways to synthesize vitamin C. Yeasts synthesize D-erythroascorbate from D-arabinose, whereas plants synthesize vitamin C from GDP-D-mannose and animals synthesize vitamin C from UDP-D-glucuronate [14-17]. Fig. (**1**) shows the vitamin C biosynthesis pathway in animals starting from D-glucose-1-P. 

Here, we review the data available concerning the vertebrate species able or unable to synthesize their vitamin C as well as the genetic basis and implications of the repeated losses and gains that have been observed.

## VITAMIN C LOSS IN TELEOST FISHES

It was originally believed that all fish species lacked the ability to synthesize ascorbic acid and that vitamin C synthesis had first appeared in amphibians [7, 18]. There have also been studies which claimed that some teleost fish species could synthesize vitamin C and a study that claimed that some non-teleost fishes could not synthesize vitamin C [19-23]. However, it has now been established that all cartilaginous and non-teleost bony fish species are able to synthesize vitamin C and that no teleost fish species can do so (references [24, 25], Fig. **2**, Supplemental Table **1**). The ability to synthesize vitamin C has been observed in hagfish, lampreys, sharks, rays, lungfish, coelacanths, sturgeons and bowfins, among others, but never in any of the numerous teleost fish species which have been studied (Fig. **2**, Supplemental Table **1**). This demonstrates that vitamin C synthesis is an ancestral trait of vertebrates and that it was lost in the common ancestor of teleost fish (reference [26], Fig. **2**). Since several ancestral actinopterygian fish species synthesize vitamin C, this mutation must have occurred some 200 to 210 MYA [24, 27]. 

**Fig. (1):**
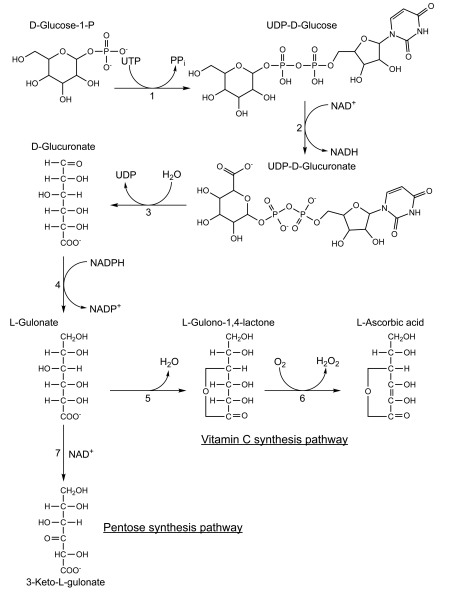
Biochemical pathway of vitamin C synthesis in vertebrates. Numbers represent the following enzymes: 1. UDP-glucose pyrophosphorylase (EC 2.7.7.9), 2. UDP-glucose dehydrogenase (EC 1.1.1.22), 3. UDP-glucuronidase (EC 3.2.1.31), 4. Glucoronate reductase (EC 1.1.1.19), 5. Gluconolactonase (EC 3.1.1.17), 6. L-gulonolactone oxidase (GLO, EC 1.1.3.8), 7. L-gulonate 3-dehydrogenase (EC 1.1.1.45). This figure is based on information from references [16, 56].

**Fig. (2):**
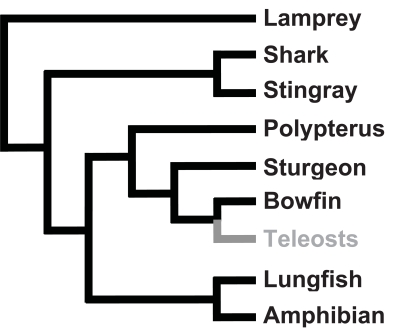
Phylogenetic distribution of the ability to synthesize vitamin C in cartilaginous and bony fishes. Lineages able to synthesize vitamin C are in black, those incapable are in gray. The phylogenetic relationships are based on those in references [24, 26]. The complete species list, species names and references are given in Supplemental Table **1**.

This loss is likely due do the complete loss of the *GLO* gene because BLAST searches cannot identify *GLO* gene sequences in any of the completely sequenced teleost fish genomes [28]. These negative results are likely significant because BLAST searches using a chicken GLO protein sequence (GenBank accession number XP_001234314) as a query readily finds numerous GLO protein sequences, including that of the sturgeon species *Acipenser transmontanus* (GenBank accession number ABO15549, which is 74% identical to that of chicken) and even that of the tunicate *Ciona intestinalis* (GenBank accession number XP_ 002122023, which is 48% identical to that of chicken). Given the high degree of conservation of this gene, the fact that *GLO* orthologues cannot be identified from the currently available teleost genomes is likely the result of the fact that this non-functional gene has mutated beyond recognition or that it has been deleted from teleost genomes altogether. Furthermore, the gene coding for gulonolactonase, the penultimate enzyme in vitamin C synthesis (Fig. **1**), is readily identified in BLAST searches using the human protein as a query (GenBank accession number EAL24133) in teleost genomes such as that of the zebra fish *Danio rerio* (GenBank accession number AAI52248), the salmon *Salmo salar* (GenBank accession number ACI69713) and the European flounder *Platichthys flesus* (GenBank accession number ACI69713), among others. Again, this suggests that the fact that *GLO* gene sequences cannot be found in any of the completely sequenced teleost fish genomes is due to their absence from these genomes and not to the methods that are used to find them.

## VITAMIN C LOSS IN ANTHROPOID PRIMATES AND GUINEA PIGS

Fig. (**3**) shows that vitamin C synthesis is also an ancestral trait of mammals and that this trait has been lost in three mammalian lineages: bats, guinea pigs and anthropoid primates (Supplemental Table **2**). In contrast with teleosts fish species, the genomes of anthropoid primates and guinea pigs still contain sequences similar to *GLO* gene sequences, although they are highly mutated and do not code for a functional L-gulono-γ-lactone oxidase protein [29-31]. The *GLO* gene of anthropoid primates has lost seven of the twelve exons found in functional vertebrate *GLO* genes, whereas the guinea pig has lost its first and fifth exon as well as part of its sixth exon (references [29, 30], Fig. **4**). Using comparison between functional and non-functional sequences, the inactivation dates have been calculated to be about 61 MYA in anthropoid primates and 14 MYA in guinea pigs [28]. Given current knowledge of vertebrate species divergence times, these estimates are consistent with those obtained based on the phylogenetic distribution of functional and non-functional *GLO* genes [29-34].

The nature of the initial mutations which inactivated the anthropoid primate and guinea pig *GLO* genes is not known. The fact that exons are missing in both cases, together with the observation that repetitive elements are often involved in exon loss events [35], suggest that recombination between repetitive elements could have been involved. However, studies which investigated this possibility concluded that such events were unlikely to have been involved in the inactivation of the anthropoid primate and guinea pig *GLO* genes [28, 36]. 

## VITAMIN C LOSS IN BATS

**Fig. (3):**
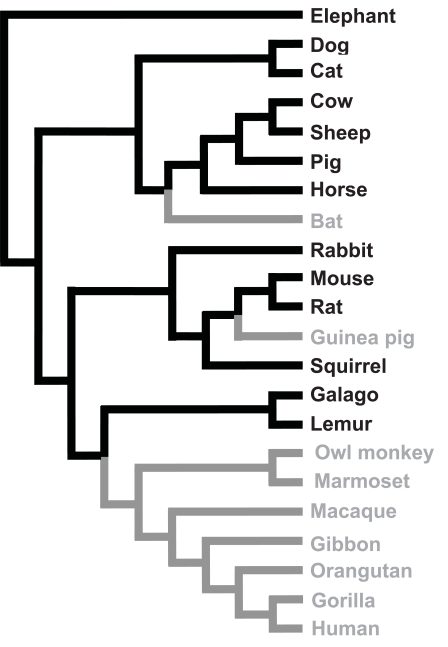
Phylogenetic distribution of the ability to synthesize vitamin C in mammals. Lineages able to synthesize vitamin C are in black, those incapable are in gray. The phylogenetic relationships are based on those in reference [63]. The complete species list, species names and references are given in Supplemental Table **2**.

**Fig. (4):**
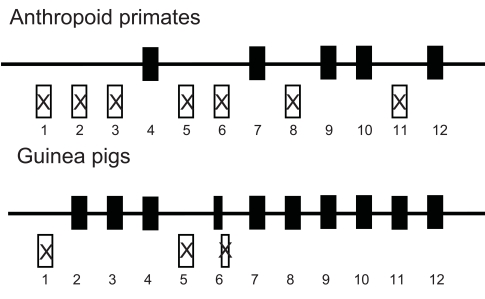
Schematic representations (not to scale) of the *GLO* gene structure in anthropoid primates and in guinea pigs. Black boxes represent exons that are still found in the genome of these species whereas white boxes with an X represent deleted exons or exon parts. Numbering refers to the exon numbers.

**Fig. (5):**
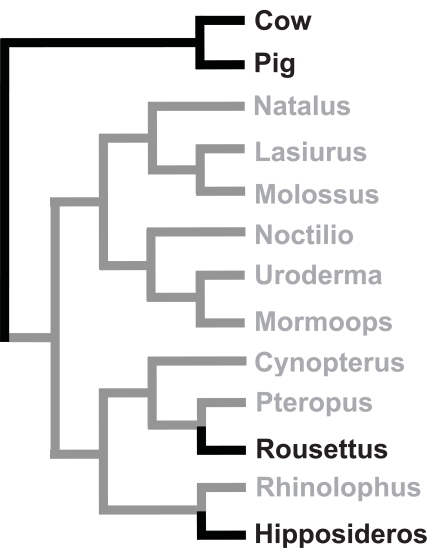
Phylogenetic distribution of the ability to synthesize vitamin C in bats. Lineages able to synthesize vitamin C are in black, those incapable are in gray. The phylogenetic relationships are based on those in reference [64]. The complete species list, species names and references are given in Supplemental Table **3**.

Following the reports that two bat species, *Pteropus sp* and *Vesperugo abramus* from two different suborders (Megachiroptera and Microchiroptera, respectively) were unable to synthesize vitamin C [4, 6], Birney *et al*. [37] assayed the presence of the GLO protein in the liver of 34 bats species from 6 different Chiroptera families. Since they did not detect GLO activity in any of these species, they concluded that all bats lacked the ability to synthesize vitamin C (Fig. **3**). However, Cui *et al*. [38] recently sequenced *GLO* cDNAs from the bats *Rousettus leschenaultia* (a Megachiroptera species, GenBank accession number HQ415789) and *Hipposideros armiger* (a Microchiroptera species, GenBank accession number HQ415790) and showed these genes produced functional GLO proteins in both species. However, their expression levels were 6-fold and 4-fold less than those of mice, in *R. leschenaultia* and *H. armiger*, respectively. Note that the GLO activity in these two species had not been assessed previously [37]. Given the currently accepted phylogeny of bats, these results therefore conclusively demonstrate that inactive genes can be reactivated during evolution (Fig. **5**, Supplemental Table **3**). Such reactivation is made possible by the fact that the sequence of these two *GLO* genes is very well conserved relative to that of other mammalian species (Fig. **5** of reference [38]). Therefore, reactivation likely only requires mutations in sites involved in the regulation of the expression of these genes. The fact that the activity level of these two genes is less than that of mice suggests that additional mutations would be necessary to further increase their expression. Alternatively, since many other bat species do not require a functional *GLO* gene, these two genes may evolve to become pseudogenes (reference [38] and see below).

Interestingly, Cui *et al*. [38] were also able to identify exons 3-8 and exons 11 and 12 from the sequence of the megabat genome *Pteropus vampyrus*, a genus where GLO activity had previously been shown to be absent (see above). The absence of indels or stop codons in these coding sequences suggests that the structure of this gene is intact. However, the analysis of the putative amino acid sequence of this gene showed that it evolves under relaxed selective constrains and has accumulated 8 amino acid changes at sites which are perfectly conserved in 11 other mammalian species (Fig. **5** of reference [38]). Therefore, even if this gene is expressed (which the authors did not assess because they did not have access to tissues for this species), it would likely not code for a functional protein [38]. Given the nature of the mutations found in the gene of this species, it could represent an example of a gene which could not be reactivated during evolution because too many reversions would be required.

## VITAMIN C LOSS IN BIRDS

The first extensive study of the capacity to synthesize vitamin C in birds showed that whereas most bird species studied were able to synthesize vitamin C, the organ where it was synthesized varied [4]. It was synthesized in the kidneys of 7 species, the kidneys and liver of 2 species, the liver of 5 species and by neither organ in one species. Because the species where vitamin C synthesis was either absent or only found in the liver were all Passeriformes birds, an evolutionarily more recent taxon, the authors interpreted their data as reflecting an “evolutionary ascent” where vitamin C synthesis went from the kidney to the liver and then disappeared altogether [4]. 

**Fig. (6):**
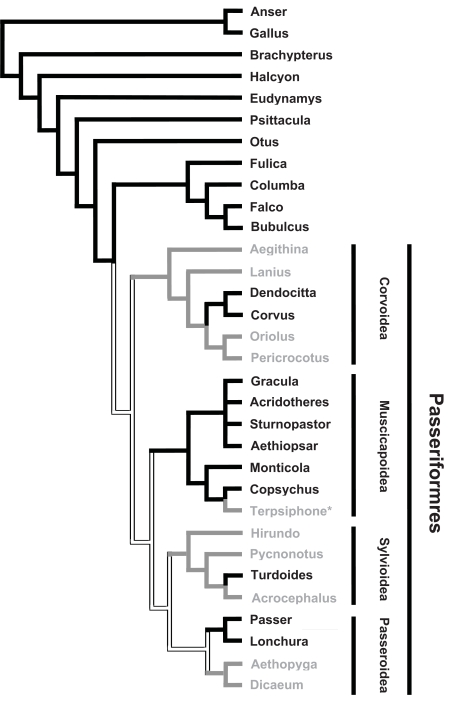
Phylogenetic distribution of the ability to synthesize vitamin C in birds. Lineages able to synthesize vitamin C are in black, those incapable are in gray and empty branches represent ancestral lineages where the status of vitamin C production is not known. The phylogenetic relationships are based on those in reference [40] and the start (*) next to the Terpsiphone genus indicates that it is now known to belong to the Corvidea family. The complete species list, species names and references are given in Supplemental Table **4**.

A subsequent study reported that 15 other Passeriformes bird species also lacked the ability to synthesize vitamin C [5]. Here again, these authors interpreted their data as showing an evolutionary progression form synthesis in the kidneys, to synthesis in both the kidneys and liver, to synthesis solely in the liver and then to the complete loss of the ability to synthesize vitamin C. To them this inability, which is also found in anthropoid primates, reflected the fact that these birds were “more evolved”. However, a phylogenetic reanalysis of Ray Chaudhuri and Chatterjee’s [5] data, based on the bird phylogenetic relationships derived from Sibley and Ahlquist’s [39] work, revealed a very different picture (reference [40], Fig. **6**, Supplemental Table **4**). Although, this phylogeny cannot be used to determine whether the inability to synthesize vitamin C is ancestral or derived in several locations at the base of this tree, it clearly shows that Passeriformes that are unable to synthesize vitamin C are not monophyletic. If the inability to synthesize vitamin C is the assumed ancestral state, then the ability of synthesizing vitamin C has been reacquired four times and lost once. In contrast, if one assumes that the ability to synthesize vitamin C is ancestral in the Passeriformes, then the ability of synthesizing vitamin C has been reacquired three times and lost three times (reference [40], Fig. **6**). 

Recent avian phylogenetic studies support the bird phylogeny that Martinez del Rio [40] derived from Sibley and Ahlquist’s work, the only notable exception being the phylogenetic position of the *Terpsiphone* genus within the Muscicapidea superfamily (Fig. **6**). Two distinct phylogenetic studies have suggested that the *Terpsiphone* genus should not be placed within the Muscicapidea superfamily but rather as part of the Corvoidea superfamily [41-42]. This implies that birds of the *Terpsiphone* genus lost their functional *GLO* gene with the last common ancestor of the Corvoidea superfamily rather than independently, removing one *GLO* gene loss from both alternative hypotheses proposed by Martinez del Rio [40].

Apart from the phylogenetic position of the *Terpsiphone* genus just mentioned, the phylogenetic relationships within the Corvoidea superfamily shown in Fig. (**6**) have been supported by recent studies [41, 43-46]. Interestingly, the phylogenetic position of the *Corvus* and *Dendrocitta* genera strongly supports the inference that reactivation of the *GLO* gene occurred in their common ancestor (Fig. **6**). 

Much like the Corvoidea superfamily, the Passeroidea superfamily relationships shown in Fig. (**6**) have been supported by recent phylogenetic studies. This superfamily is therefore composed of two different groups: one containing the *Passer* and *Lonchura *genera which can synthesize vitamin C and a second group containing *Aethopyga* and *Dicaeum* who cannot [44, 47, 48]. Although, these two different groups clearly share a common ancestor, the absence of information regarding the ancestral state of Passeriformes does not allow one to determine whether this represents an additional reacquisition or loss of a functional *GLO* gene. 

The position of the *Turdoides* genus within the Sylvioidea superfamily is supported by current studies. Although, the phylogenetic position of this genus has not yet been investigated in detail, it is closely related to both the *Garrulax* [49-51] and the *Yuhina* genera [49] which have both been shown to be part of the Sylvioidea superfamily [47]. The monophyletic nature of the Sylvioidea superfamily is also supported by current data [52]. Furthermore, within the Sylvioidea superfamily, the phylogenetic positions of the *Acrocephalus* and the *Pycnonotus* genera, relative to the *Turdoides* genus, are supported by recent studies [42, 47, 49, 51-55]. Here again, this strongly supports the inference that the ability to synthesize vitamin C has been reacquired in the *Turdoides* genus (reference [40], Fig. **6**). 

In conclusion, given the set of Passeriformes bird species currently known to be able and unable to synthesize vitamin C and the currently accepted phylogenetic relationships of these species, one can deduce that the ability of synthesizing vitamin C has been unequivocally reacquired at least twice, once in the *Corvus*/*Dendrocitta* lineage and once in the *Turdoides* lineage. If one assumes that the inability to synthesize vitamin C is ancestral in the Passeriformes, then the ability of synthesizing vitamin C has been reacquired four times. If one assumes that the ability to synthesize vitamin C is ancestral in the Passeriformes, then the ability of synthesizing vitamin C has been reacquired three times and lost twice. 

## WHY ARE MUTATIONS LIMITED TO *GLO* GENES?

Given its high daily requirement and important functions, it is surprising that many species, such as teleost fishes, anthropoid primates, guinea pigs, as well as some bat and Passeriformes bird species, have lost the capacity to synthesize it. Interestingly, all the known losses are the result of mutations in the L-gulono-γ-lactone oxidase gene (*GLO*; EC number 1.1.3.8) which codes for the enzyme that catalyzes the final step of vitamin C biosynthesis (Fig. **1**). Therefore, losing this gene only impacts the ability to make vitamin C (Fig. **1**, references [16, 56]). In contrast, losing genes for other enzymes in this synthetic pathway would affect the production of many other molecules (Fig. **1**, reference [16]). For example, losing the gene coding for gluconolactonase (EC number 3.1.1.17), would not only affect the formation of L-gulono-1,4-lactone, but would also affect caprolactam degradation and the pentose phosphate pathway, among others [57]. Compared to other genes, the *GLO* gene is therefore “predisposed” to being lost because it makes a single compound unnecessary for other pathways.

## EVOLUTIONARY IMPLICATIONS

Explaining the frequent loss of *GLO* genes by saying that it only affects the production of a single compound also implies that losing the capacity to make this compound is not selected against, i.e., that such a loss does not cause any selective disadvantage. Since all species which have lost the capacity to synthesize vitamin C have a vitamin C-rich diet, this is the most common explanation brought forward to explain its frequent occurrence [8, 28, 29, 37, 58]. This explanation is consistent with the fact that wild anthropoid primates (unable to synthesize vitamin C) consume much more vitamin C than the recommended daily allowance for adult humans in the USA, about 1 mg/kg/day. For example, gorillas (*Gorilla gorilla*) consume 20-30 mg/kg/day, howler monkeys (*Alouatta palliata*) consume 88 mg/kg/day, and spider monkeys (*Ateles geoffroyi*) consume 106 mg/kg/day [58]. The same is true with bats, with *Artibeus jamaicensis* consuming 258 mg/kg/day [58]. Although, minimum daily requirements have not been established for these wild species, it seems reasonable to assume that they obtain ample vitamin C supplies from their diets. 

Another argument supporting the suggestion that species which have lost their *GLO* gene were under no selective pressure to keep it, is that all species which have lost their *GLO* gene have very different diets but all of them have diets rich in vitamin C [59]. The diets of *GLO*-less anthropoid primate species contain plants that are rich in vitamin C. Those of bats include species whose primary foods are fish, fruit, pollen and nectar, blood or insects [37]. The same is also true of bird species where species having a particular diet are just as likely to be or not to be able to synthesize their vitamin C as long as this diet is rich in vitamin C (Table **1**). The converse is also true: no species lacking GLO activity has ever been found to have a diet poor in vitamin C, such as a diet composed uniquely of seeds (reference [37], Table **1**). Finally, although it has been suggested that a regular diet does not contain enough vitamin C, experiments with guinea pigs did not detect any beneficial effects by increasing the amount of vitamin C in the diet of these animals [7, 8, 60]. 

Interestingly, the loss of genes coding for essential compounds is not limited to animals. Recent work has shown that numerous algae species, which are often assumed to be autotrophic, are in fact auxotrophic for vitamins B_1_, B_7_ and B_12_. Although, the requirement for vitamin B_12_ is the result of the absence of the complete biosynthetic pathway for this compound in algae (it is only present in bacteria), the requirements for vitamins B1 and B7 are due to the loss of the genes coding for one or more key biosynthetic enzymes [61]. Here again, because these vitamins are available from other sources, these losses are thought not to have been selected against [61].

In vertebrates, there also does not seem to be strong selective pressure to regain lost GLO activity. In bats, *R. leschenaultia* is a frugivorous species with a resuscitated *GLO* gene which is closely related to *C. sphinx*, another frugivorous species, but incapable of synthesizing vitamin C. Similarly, *H. armiger* is an insectivorous species with another resuscitated *GLO* gene which is closely related to another insectivorous species, *Rhinolophus ferrumequinum*, which cannot synthesize vitamin C [38]. The fact that these two pairs of species have similar diets, and that the expression level of these *GLO* genes is low compared to that of mice (see above), led Cui *et al*. [38] to suggest that these two genes will, in the future, become pseudogenes and that the two species with the resuscitated *GLO* genes will lose the ability to synthesize vitamin C. The same situation might be present in birds because the two unambiguous reactivation events, that in *Corvus/Dendrocitta* and that in *Turdoides*, involve species which are closely related to species with similar diets but which cannot synthesize vitamin C (Table **1**). The fact that reactivations occur in species which have already ample supplies of vitamin C in their diets suggests that these reactivations are random and not selected for.

An alternative hypothesis is that losing the capacity to synthesize vitamin C might be advantageous because vitamin C synthesis leads to the formation of hydrogen peroxide (H_2_O_2_) and the depletion of glutathione (Fig. **1**, reference [62]). However, if this hypothesis were true, then *GLO* gene reactivations would be selected against. Since *GLO* gene reactivations have been documented in bat and bird species, this hypothesis is no longer tenable. Therefore, current evidence favors the hypothesis that the multiple gains and losses in the ability to synthesize vitamin C are random, as would be expected for a neutral trait. 

The neutrality of vitamin C loss is a function of the environment in which species lives. Individuals from a species which have lost the ability to make their own vitamin C will not be selected against as long as their diet contains sufficient quantities of vitamin C. This is why the function of the *GLO* gene has been lost repeatedly during evolution. However, individuals from such a species will develop scurvy if their diet changes so as to no longer contain sufficient amounts of vitamin C. Such diet changes occurred repeatedly during human history, causing the death of millions of people [65, 66]. Vitamin C loss is therefore only neutral when sufficient quantities of vitamin C are part of the diet.

## SUPPLEMENTARY MATERIAL

Supplementary material is available on the publishers Web site along with the published article.

## Figures and Tables

**Table 1:** Diets of Passeriformes Genera

Superfamily	Genus	Vitamin C Synthesis?	Diet
Corvoidea	*Oriolus*	No	Insectivores and frugivores
Corvoidea	*Pericrocotus*	No	Insectivores
Corvoidea	*Lanius*	No	Insectivores and carnivores of birds, reptiles and mammals
Corvoidea	*Aegithina*	No	Insectivores
Corvoidea	*Corvus*	Yes	Carnivores, insectivores, granivores and some scavengers
Corvoidea	*Dendrocitta*	Yes	Insectivores, frugivores
Muscicapoidea	*Terpsiphone*	No	Insectivores
Sylvloidea	*Acrocephalus*	No	Insectivores and frugivores
Sylvloidea	*Pycnonotus*	No	Insectivores, frugivores and nectarivores
Sylvloidea	*Hirundo*	No	Insectivores
Sylvloidea	*Turdoides*	Yes	Frugivores, insectivores and carnivores
Passeroidea	*Aethopyga*	No	Nectarivores
Passeroidea	*Dicaeum*	No	Insectivores, frugivores and nectarivores
Passeroidea	*Passer*	Yes	Granivores, insectivores, and some omnivores
Passeroidea	*Lonchura*	Yes	Herbivores (Algae)
