# Supplementary material for: The Genetics of Vitamin C Loss in Vertebrates
Source: Curr Genomics. 2011 Aug;12(5):371–8. doi: 10.2174/138920211796429736 (PMC3145266; doi:10.2174/138920211796429736)
Supplement: Supplementary file 1 [file CG-12-371_SD1.pdf]

SUPPORTIVE/SUPPLEMENTARY MATERIAL

Supplemental Table 1. Fish and other Non-Mammalian Vertebrate Species with/without the Capacity to Make Vitamin C

Species	Family	Order	Common name	Vitamin C production	Organ	References
Petromyzon marinus	Petromyzontidae	Petromyzontiformes	Sea lamprey	Yes	Kidney	[1]
Lampetra japonica	Petromyzontidae	Petromyzontiformes	Japanese lamprey	Yes	Kidney	[2]
Triakis scyllium	Triakidae	Carcharhiniformes	Banded houndshark	Yes	Kidney	[2, 3]
Mustelus manazo	Triakidae	Carcharhiniformes	Starspotted smooth-hound shark	Yes	Kidney	[2, 3]
Mustelus griseus	Triakidae	Carcharhiniformes	Japanese gray smooth hound shark	Yes	Kidney	[3]
Raja kenojei	Rajidae	Rajiformes	Ocellate spot skate	Yes	Kidney	[2, 3]
Dasyatis akajei	Dasyatidae	Myliobatiformes	Red stingray	Yes	Kidney	[2, 3]
Potamotrygon sp C.	Potamotrygonidae	Myliobatiformes	Freshwater stingray	Yes	Kidney	[4]
Acipenser baeri	Acipenseridae	Acipenseriformes	Siberian sturgeon	Yes	Kidney	[3]
Acipenser guelden-staedtii	Acipenseridae	Acipenseriformes	Russian sturgeon	Yes	Kidney	[3]
Acipenser naccarii	Acipenseridae	Acipenseriformes	Adriatic sturgeon	Yes	Kidney	[3]
Acipenser transmon-tanus	Acipenseridae	Acipenseriformes	White sturgeon	Yes	Kidney	[3, 5, 9]
Acipenser ruthenus	Acipenseridae	Acipenseriformes	Sterlet	Yes	Kidney	[3, 6]
Scyliorhinus torazame	*Scyliorhinidae*	Carcharhiniformes	Cloudy catshark	Yes	Kidney	[3]
Squalus acanthias	Squalidae	Squaliformes	Picked dogfish	Yes	Kidney	[3, 6]
Polyodon spathula	Polyodontidae	Acipenseriformes	Paddlefish	Yes	Kidney	[5]
Protopterus aethio-picus	Ceratodontidae	Ceratodontiformes	African lungfish	Yes	Kidney	[2]
Neoceratodus for-steri	Ceratodontidae	Ceratodontiformes	Australian lungfish	Yes	Kidney	[7]
Lepidosiren para-doxa	Lepidosirenidae	Lepidosireniformes	South African lungfish	Yes	Kidney	[4]
Polypterus senegalus	Polypteridae	Polypteriformes	*Polypterus*	Yes	Kidney	[8]
Amia calva	Amiidae	Amiiformes	Bowfin	Yes	Kidney	[8]
Lepisosteus osseus	Lepisosteidae	Lepisosteiformes	Longnose gar	Yes	Kidney	[8]
Carassius auratus	Cyprinidae	Cypriniformes	Goldfish	No	n.a.	[8]
Cyprinus carpio	Cyprinidae	Cypriniformes	Common carp	No	n.a.	[8]
Oncorhynchus mykiss	Salmonidae	Salmoniformes	Rainbow trout	No	n.a.	[9]
Ictalurus punctatus	Ictaluridae	Siluriformes	Catfish	No	n.a.	[9]
Oryzias latipes	Adrianichthyidae	Beloniformes	Medaka	No	n.a.	[10]

(Table S1). Contd…..

Species	Family	Order	Common name	Vitamin C production	Organ	References
Osteoglossum bicirrhosum	Osteoglossidae	Osteoglossiformes	Arowana	No	n.a.	[4]
Pelona sp.	Clupeidae	Clupeiformes	Sardine	No	n.a.	[4]
Arapaima gigas	Osteoglossidae	Osteoglossiformes	Arapaima	No	n.a.	[4]
Pygocentrus nattereri	Characidae	Characiformes	Piranha caju	No	n.a.	[4]
Serrasalmus elongatus	Characidae	Characiformes	Piranha mucura	No	n.a.	[4]
Schizodon fascaitus	Characidae	Characiformes	Aracu comum	No	n.a.	[4]
Colossoma macropomum	Characidae	Characiformes	Tambaqui	No	n.a.	[4]
Hypostomus sp.	Loricariidae	Siluriformes	Acari-pedra	No	n.a.	[4]
Steatogenys elegans	Hypopomidae	Gymnotiformes	Sarapo	No	n.a.	[4]
Electrophorus electricus	Gymnotidae	Gymnotiformes	Electric eel	No	n.a.	[4]
Cichla sp.	Cichlidae	Perciformes	Peacock bass	No	n.a.	[4]
Clupea harengus	Clupeidae	Clupeiformes	Atlantic herring	No	n.a.	[6]
Anguilla anguilla	Anguillidae	Anguilliformes	European eel	No	n.a.	[6]
Salmo salar	Salmonidae	Salmoniformes	Atlantic salmon	No	n.a.	[6]
Gadus morhua	Gadidae	Gadiformes	Atlantic cod	No	n.a.	[6]
Scomber scombrus	Scombridae	Perciformes	Atlantic mackerel	No	n.a.	[6]
Hippoglossus hippoglossus	Pleuronectidae	Pleuronectiformes	Atlantic halibut	No	n.a.	[6]
Scophthalmus maximus	Scophthalmidae	Pleuronectiformes	Atlantic turbot	No	n.a.	[6]
Rutilus rutilus	Cyprinidae	Cypriniformes	Common roach	No	n.a.	[11]
Coregonus lavaretus	Salmonidae	Salmoniformes	Common whitefish	No	n.a.	[11]
Salvelinus alpinus	Salmonidae	Salmoniformes	Arctic char	No	n.a.	[11]
Rana tigrina	Ranidae	Anura	Indus valley bullfrog	Yes	Kidney	[12]
Testudo elegans	Testudinidae	Testudines	Tortoise	Yes	Kidney	[12]
Lissemys punctata	Trionychidae	Testudines	Indian flap-shelled turtle	Yes	Kidney	[12]
Bufo melanostictus	Bufonidea	Anura	Asian common toad	Yes	Kidney	[12, 13]
Mabuya carinata	Scincidae	Squamata	Keeled Indian Mabuya	Yes	Kidney	[12]
Natrix piscator	Colubridae	Squamata	Snake	Yes	Kidney	[12]
Hemidactylus flaviviridis	Gekkonidae	Squamata	Yellow-bellied House Gecko	Yes	Kidney	[12]
Calotes versicolor	Agamidae	Squamata	Oriental garden lizard	Yes	Kidney	[12, 13]
Varanus monitor	Varanidae	Squamata	Common Indian Monitor	Yes	Kidney	[12, 13]

[1] Moreau, R.; Dabrowski, K. Body pool and synthesis of ascorbic in adult sea lamprey (*Petromyzon marinus*): An agnathan fish with gulonolactone oxidase activity. *Proc. Natl. Acad. Sci. U.S.A.*, **1998**, *95*, 10279-10282.
[2] Touhata, K.; Toyohara, H.; Mitani, T.; Kinoshita, M.; Satou, M.; Sakaguchi, M. Distribution of L-Gulono-1, 4-Lactone Oxidase among Fishes. *Fish. Sci.*, **1995**, *61*, 729-730.

(Table S1). Contd…..

[3] Cho, Y. S.; Douglas, S. E.; Gallant, J. W.; Kim, K.Y.; Kim, D. S.; Nam, Y. K. Isolation and characterization of cDNA sequences of L-gulono-gamma-lactone oxidase, a key enzyme for biosynthesis of ascorbic acid, from extant primitive fish groups. *Comp. Biochem. Physiol. B Biochem. Mol. Biol.*, **2007**, *147*, 178-190.
[4] Fracalossi, D.M.; Allen, M.E.; Yuyama, L.K.; Oftedal, L.K. Ascorbic acid biosynthesis in Amazonian fishes. *Aquaculture,* **2001**, *192*, 321-332.
[5] Dabrowski, K. Primitive Actinopterigian fishes can synthesize ascorbic acid. *Experientia*, **1994**, *50*, 745-748.
[6] Maeland, A.; Waagbø, R. Examination of the qualitative ability of some cold water marine teleosts to synthesize ascorbic acid. *Comp Biochem Physiol A Mol Integr Physiol.*, **1998**, *121*, 249-255.
[7] Dykhuizen, D.E.; Harrison, K.M.; Richardson, B.J. Evolutionary implications of ascorbic acid production in the Australian lungfish. *Experientia*, **1980**, *36*, 945-946.
[8] Moreau, R.; Dabrowski, K. Biosynthesis of ascorbic acid by extant actinopterigians. *Journal of Fish Biology*, **2000**, 57, 733-745.
[9] Moreau, R.; Dabrowski, K. The primary localization of ascorbate and its synthesis in the kidneys of acipenserid (Chondrostei) and teleost (Teleostei) fishes. *J. Comp. Physiology*, **1996**, *166*, 178-183.
[10] Toyohara, H.; Nakata, T.; Touhata, K.; Hashimoto, H.; Kinoshita, M.; Sakaguchi, M.; Nishikimi, M.; Yagi, K.; Wakamatsu, Y.; Ozato, K. Transgenic Expression of L-Gulono-g-lactone Oxidase in Medaka (Oryzias latipes), a Teleost Fish That Lacks This Enzyme Necessary for L-Ascorbic Acid Biosynthesis. *Biochem. And Biophy. Res. Com.*, **1996**, *223*, 650-653.
[11] Dabrowski, K. Ascorbate concentration in fish ontogeny. *J. Fish Biol.*, **1992**, *40*, 273-279.
[12] Chatterjee, I.B. Evolution and the biosynthesis of ascorbic acid. *Science*, **1973**, *182*, 1271-1272.
[13] Roy, R.N.; Guha, B.C. Species difference in regard to the biosynthesis of ascorbic acid. *Nature*, **1958**, *182*, 319-318.

Supplemental Table 2. Mammalian Species with/without the Capacity to Make Vitamin C

Species	Family	Order	Common name	Vitamin C production	Organ	References
Tachyglossus aculeatus	Tachyglossidae	Monotremata	Australian echidna	Yes	Kidney	[1]
Ornithorhynchus anatinus	Ornithorhynchidae	Monotremata	Platypus	Yes	Kidney	[1]
Didelphis virginiana	Didelphidae	Didelphimorphia	North American opossum	Yes	Liver	[1]
Dasyuroides byrnei	Dasyuridae	Dasyuromorphia	Kowari	Yes	Liver	[1]
Antechinus stuartii	Dasyuridae	Dasyuromorphia	Brown antechinus	Yes	Liver	[1]
Dasyurus maculatus	Dasyuridae	Dasyuromorphia	Spotted-tailed quoll	Yes	Liver	[1]
Perameles nasuta	Peramelidae	Peramelemorphia	Long-nosed bandicoot	Yes	Liver & kidney	[1]
Isoodon macrourus	Peramelidae	Peramelemorphia	Northern brown bandicoot	Yes	Liver & kidney	[1]
Pseudocheirus peregrinus	Pseudocheiridae	Diprotodontia	Common ring-tailed possum	Yes	Liver	[1]
Schoinobates volans	Petauridae	Diprotodontia	Greater Gliding Possum	Yes	Liver	[1]
Cercartetus nanus	Burramyidae	Diprotodontia	Dormouse Possum	Yes	Liver	[1]
Vombatus ursinus	Vombatidae	Diprotodontia	Common wombat	Yes	Liver	[1]
Trichosurus vulpecula	Phalangeridae	Diprotodontia	Common brush-tailed possum	Yes	Liver	[1]
Macropus rufogriseus	Macropodidae	Diprotodontia	Red-necked wallaby	Yes	Liver	[1]
Macropus eugenii	Macropodidae	Diprotodontia	Tammar wallaby	Yes	Liver	[1]
Macropus giganteus	Macropodidae	Diprotodontia	eastern gray kangaroo	Yes	Liver	[1]
Macropus robustus	Macropodidae	Diprotodontia	Wallaroo	Yes	Liver	[1]
Thylogale thetis	Macropodidae	Diprotodontia	Red-necked pademelon	Yes	Liver	[1]
Wallabia bicolor	Macropodidae	Diprotodontia	Swamp wallaby	Yes	Liver	[1]

(Table S2). Contd…..

Species	Family	Order	Common name	Vitamin C production	Organ	References
Myoprocta acouchy	Dasyproctidae	Rodentia	Acouchi	Yes	Liver	[2]
Dasyprocta aguti	Dasyproctidae	Rodentia	Brazilian agouti	Yes	Liver	[2]
Cavia porcellus	Caviidae	Rodentia	Guinea pig	No	n.a.	[3, 4, 5, 6, 7]
Canis familiaris	Canidae	Carnivora	Dog	Yes	Liver	[3, 6, 8]
Felis catus	Felidae	Carnivora	Cat	Yes	Liver	[3, 6]
Sus scrofa	Suidae	Artiodactyla	Pig	Yes	Liver	[8]
Bos primigenius	Bovidae	Artiodactyla	Cow	Yes	Liver	[3, 6]
Capra hircus	Bovidae	Artiodactyla	Goat	Yes	Liver	[9]
Ovis aries	Bovidae	Artiodactyla	Sheep	Yes	Liver	[3, 6]
Oryctolagus cuniculus	Leporidae	Lagomorpha	Rabbit	Yes	Liver	[3]
Sciurus carolinensis	Sciuridae	Rodentia	Grey squirrel	Yes	Liver	[3, 6, 8]
Mus musculus	Muridae	Rodentia	Mouse	Yes	Liver	[3, 5, 6, 8]
Rattus norvegicus	Muridae	Rodentia	Rat	Yes	Liver	[3, 4, 5, 6, 8]
?	Muridae	Rodentia	Gerbil	Yes	Liver	[6, 9]
Homo sapiens	Hominidae	Primates	Human	No	n.a.	[3, 4, 6]
Macaca mulatta	Cercopithecidae	Primates	Macaque	No	n.a.	[3, 6]
Chlorocebus aethiops	Cercopithecidae	Primates	African green monkey	No	n.a.	[4]
Tarsius bancanus	Tarsiidae	Primates	Horsfield's tarsier	Yes	Liver & kidney*	[5]
Microcebus murinus	Cheirogaleidae	Primates	Gray mouse lemur	Yes	Liver & kidney*	[5]
Cheirogaleus medius	Cheirogaleidae	Primates	Lesser dwarf lemur	Yes	Liver & kidney*	[5]
Propithecus verreauxi	Indriidae	Primates	White sifaka	Yes	Liver & kidney*	[5]
Galago senegalensis moholi	Galagidae	Primates	South African galago	Yes	Liver & kidney*	[5]
Galago garnetti	Galagidae	Primates	Small-eared galago	Yes	Liver & kidney*	[5]
Galago crassicaudatus	Galagidae	Primates	Thick-tailed bush baby	Yes	Liver & kidney*	[5]
Loris tardigradus	Lorisidae	Primates	Slender loris	Yes	Liver & kidney*	[5]
Perodicticus potto	Lorisidae	Primates	Potto	Yes	Liver & kidney*	[5]
Hapalemur griseus	Lemuridae	Primates	Bamboo lemur	Yes	Liver & kidney*	[5]
Varecia variegata	Lemuridae	Primates	Ruffed lemur	Yes	Liver & kidney	[5]
Lemur fulvus albifrons	Lemuridae	Primates	Lemur	Yes	Liver & kidney	[5]
Lemur fulvus collaris	Lemuridae	Primates	Lemur	Yes	Liver & kidney	[5]
Lemur fulvus rufus	Lemuridae	Primates	Lemur	Yes	Liver & kidney	[5]
Lemur macaco	Lemuridae	Primates	Lemur	Yes	Liver & kidney	[5]

Note. Stars (*) denote species for which GLO activity in the liver is still uncertain because the detection method used could not detect zero GLO activity.

[1] Birney, E.C.; Jenness, R.; Hume, I.D. Evolution of an enzyme system: ascorbic acid biosynthesis in monotremes and marsupials. *Evolution*, **1980**, *34*, 230-239.

(Table S2). Contd…..

[2] Yess, N.J.; Hegsted, D.M. Biosynthesis of ascorbic acid in the acouchi and agouti. *J. Nutr*., **1967**, *92*, 331-333.
[3] Chatterjee, I.B.; Majumder, A.K.; Nandi, B.K.; Subramanian, N. Synthesis and some major functions of vitamin c in animals. *Ann. N. Y. Acad. Sci*., **1975**, *258*, 24-47.
[4] Sato, P.; Udenfriend, S. Scurvy-prone animals, including man, monkey, and guinea pig, do not express the gene for gulonolactone oxidase. *Arch. Biochem. Biophys*., **1978**, *187*, 158-162.
[5] Pollock, J.I.; Mullin, R.J. Vitamic biosynthesis in Prosimians: evidence for the anthropoid affinity of tarsius. *Am. J. Phys. Anthropol*., **1987**, *73*, 65-70.
[6] Dutta Gupta, S.; Choudhury, P.K.; Chatterjee I.B. Synthesis of l-ascorbic acid from d-glucurono-1,4-lactone conjugates by different species of animals. *Int. J. Biochem*., **1973**, *4*, 309-314.
[7] Nishikimi, M.; T. Kawai, T.; Yagi, K. Guinea pigs posses a highly mutated gene for l-gulono-g-lactone oxidase, the key enzyme for L-ascorbic acid biosynthesis missing in this species. *J. Biol. Chem*., **1982**, *267*, 21967-21972.
[8] Jenness, R.; Birney, E.C.; Ayaz, K.L. Variation of L-gulonolactone oxidase activity in placental mammals. *Comp. Biochem. Physiol. B Biochem. Mol. Biol*., **1980**, *67*, 195-204.
[9] Chatterjee, I.B. Evolution and the biosynthesis of ascorbic acid. *Science*, **1973**, *182*, 1271-1272.

Supplemental Table 3. Bat Species with/without the Capacity to Make Vitamin C

Species	Family	Order	Vitamin C production	References
Myotis ricketti	Vespertilionidae	Chiroptera	No	[1]
Rousettus leschenaultii	Pteropodidae	Chiroptera	Yes, in the liver	[1]
Rhinolophus ferrumequinum	Rhinolophidae	Chiroptera	No	[1]
Hipposideros armiger	Hipposideridae	Chiroptera	Yes, in the liver	[1]
Cynopterus sphinx	Pteropodidae	Chiroptera	No	[1]
Scotophilus kuhlii	Vespertilionidae	Chiroptera	No	[1]
Noctilio leporinus	Noctilionidae	Chiroptera	No	[2]
Pteronotus davyi<br>*Pteronotus parnelli*<br>*Pteronotus suapurensis*	Mormoopidae	Chiroptera	No	[2]
Mormoops megalophylla	Mormoopidae	Chiroptera	No	[2]
Micronycteris megalotis	Phyllostomatinae	Chiroptera	No	[2]
Mimon cozumelae	Phyllostomatinae	Chiroptera	No	[2]
Glossophaga soricina	Phyllostomatinae	Chiroptera	No	[2]
Carollia brevicauda	Phyllostomatinae	Chiroptera	No	[2]
Sturnira lilium<br>*Sturnira ludovici*	Phyllostomatinae	Chiroptera	No	[2]
Uroderma bilobatum	Phyllostomatinae	Chiroptera	No	[2]
Chiroderma villosum	Phyllostomatinae	Chiroptera	No	[2]
Artibeus jamaicensis<br>*Artibeus lituratus*<br>*Artibeus phaeotis*<br>*Artibeus toltecus*	Phyllostomatinae	Chiroptera	No	[2]
Desmodus rotundus	Phyllostomatinae	Chiroptera	No	[2]
Diphylla ecaudata	Phyllostomatinae	Chiroptera	No	[2]
Natalus stramineus	Natalidae	Chiroptera	No	[2]
Myotis keaysi<br>*Myotis leibii*<br>*Myotis lucifugus*<br>*Myotis velifer*<br>*Myotis riparius*	Vespertilionidae	Chiroptera	No	[2]

(Table S3). Contd…..

Species	Family	Order	Vitamin C production	References
Eptesicus furinalis<br>*Eptesicus fuscus*	Vespertilionidae	Chiroptera	No	[2]
Lasiurus ega<br>*Lasiurus intermedius*	Vespertilionidae	Chiroptera	No	[2]
Plecotus townsendii	Vespertilionidae	Chiroptera	No	[2]
Molossus ater<br>*Molossus sinaloae*	Molossidae	Chiroptera	No	[2]
Promops centralis	Molossidae	Chiroptera	No	[2]
Eumops glaucinus	Molossidae	Chiroptera	No	[2]
Pteropus medius	Pteropodidae	Chiroptera	No	[3, 4]
Vesperugo abramus	Vespertilionidae	Chiroptera	No	[4]

[1] Cui, J.; Pan, Y.H.; Zhang, Y.; Jones, G.; Zhang, S. Progressive pseudogenization: vitamin C synthesis and its loss in bats. *Mol. Biol. Evol.*, **2011**, *28*, 1025-1031.
[2] Briney,E.C.; Jenness, R.; Ayaz, K.M. Inability of bats to synthesise L-ascorbic acid. *Nature*, **1976**, *260*, 626-628.
[3] Roy, R.N.; Guha, B.C. Species difference in regard to the biosynthesis of ascorbic acid. *Nature*, **1958**, *182*, 319-318.
[4] Dutta Gupta, S.; Choudhury, P.K.; Chatterjee I.B. Synthesis of l-ascorbic acid from d-glucurono-1,4-lactone conjugates by different species of animals. *Int. J. Biochem.*, **1973**, *4*, 309-314.

Supplemental Table 4. Bird Species with/without the Capacity to Make Vitamin C

Species	Family	Order	Vitamin C production	Organ	References
Anser indicus	Anatidae	Anseriformes	Yes	Kidney	[1, 2]
Gallus gallus	Phasianidae	Galliformes	Yes	Kidney	[1, 2, 3]
Brachypterus benghalensis	Picidae	Piciformes	Yes	Liver	[1, 2]
Halcyon smyrnensis	Halcyonidae	Coraciiformes	Yes	Kidney	[1, 2, 3]
Eudynamis scolopaceus	Cuculidae	Cuculiformes	Yes	Kidney	[1, 2]
Psittacula eupatria	Psittacidae	Psittaciformes	Yes	Kidney	[1, 2]
Otus bakkamoena	Strigidae	Strigiformes	Yes	Kidney	[1, 2, 3]
Fulica atra	Rallidae	Gruiformes	Yes	Kidney	[1, 2, 3]
Columba livia	Columbidae	Columbiformes	Yes	Kidney	[1, 2, 3]
Falco jugger	Falconidae	Falconiformes	Yes	Kidney	[1, 2]
Bubulcus ibis	Ardeidae	Ciconiiformes	Yes	Kidney	[1, 2]
Aegithina tiphia	Aegithinidae	Passeriformes	No	n.a.	[1, 2]
Lanius Schach tricolor<br>*Lanius vittatus*<br>*Lanius excubitor*	Laniidae	Passeriformes	No	n.a.	[1, 2]
Dendocitta vagabunda	Corvidae	Passeriformes	Yes	Liver	[1, 2]
Corvus splendens	Corvidae	Passeriformes	Yes	Liver & kidney	[1, 2, 3]
Oriolus xanthornus	Oriolidae	Passeriformes	No	n.a.	[1, 2]
Pericrocotus flammeus	Campephagidae	Passeriformes	No	n.a.	[1, 2]
Gracula religiosa	Sturnidae	Passeriformes	Yes	Liver	[1, 2, 3]

(Table S4). Contd…..

Species	Family	Order	Vitamin C production	Organ	References
Acridotheres tristis	Sturnidae	Passeriformes	Yes	Liver & kidney	[1, 2, 3]
Sturnopastor contra	Sturnidae	Passeriformes	Yes	Liver	[1, 2]
Aethiopsar fuscus	Sturnidae	Passeriformes	Yes	Liver	[1, 2]
Monticola cinaclorhynchus	Muscicapidae	Passeriformes	Yes	Liver	[1, 2]
Copsychus saularis	Muscicapidae	Passeriformes	Yes	Liver	[1, 2, 3]
Terpsiphone paradisi	Monarchidae	Passeriformes	No	n.a.	[1, 2]
Hirundo rustica	Hirundinidae	Passeriformes	No	n.a.	[1, 2]
Pycnonotus luteolus<br>*Pycnonotus jocosus*<br>*Pycnonotus leucogenys*	Pycnonotidae	Passeriformes	No	n.a.	[1, 2]
Pycnonotus cafer	Pycnonotidae	Passeriformes	No	n.a.	[1, 2, 3]
Turdoides somervillei	Timaliidae	Passeriformes	Yes	Liver	[1, 2]
Acrocephalus slentoreus	Acrocephalidae	Passeriformes	No	n.a.	[1, 2]
Passer domesticus	Passeridae	Passeriformes	Yes	Liver	[1, 2]
Lonchura malacca	Estrildidae	Passeriformes	Yes	Liver	[1, 2, 3]
Aethopyga siparaja	Nectariniidae	Passeriformes	No	n.a.	[1, 2]
Dicaeum erythrorhynchoas	Dicaeidae	Passeriformes	No	n.a.	[1, 2]
Rhipidura albogularis	Corvoidae	Passeriformes	No	n.a.	[1, 2]
Aythya ferina	Anatidae	Anseriformes	Yes	Kidney	[2, 3]
Francolinus pondicerianus	Phasianidae	Galliformes	Yes	Kidney	[2, 3]
Acridotheres ginginianus	Sturnidae	Passeriformes	Yes	Liver	[1, 2, 3]
Crypsirina vagabunda	Corvidae	Passeriformes	Yes	Liver	[3]

Note: n.a., not applicable.

[1] Chaudhuri, C.R.; Chatterjee, I.B. L-ascorbic acid synthesis in birds: phylogenetic trend. *Science*, **1969**, *164*, 435-436.
[2] Dutta Gupta, S.; Choudhury, P.K.; Chatterjee I.B. Synthesis of l-ascorbic acid from d-glucurono-1,4-lactone conjugates by different species of animals. *Int. J. Biochem.*, **1973**, *4*, 309-314.
[3] Roy, R.N.; Guha, B.C. Species difference in regard to the biosynthesis of ascorbic acid. *Nature*, **1958**, *182*, 319-318.
